# Supplementary material for: Ca2+-activated sphingomyelin scrambling and turnover mediate ESCRT-independent lysosomal repair
Source: Nat Commun. 2022 Apr 6;13:1875. doi: 10.1038/s41467-022-29481-4 (PMC8986845; doi:10.1038/s41467-022-29481-4)

# Supplementary Information

## $Ca^{2+}$-activated sphingomyelin scrambling and turnover mediate ESCRT-independent lysosomal repair

**Authors:** Patrick Niekamp[1], Felix Scharte[2], Tolulope Sokoya[1], Laura Vittadello[3], Yeongho Kim[4], Yongqiang Deng[4], Elisabeth Südhoff[1], Angelika Hilderink[1], Mirco Imlau[2], Christopher J. Clarke[5], Michael Hensel[2], Christopher G. Burd[4], Joost C. M. Holthuis[1]*

**This PDF file includes:**

Supplementary Figs. 1 to 12

Image J Macros

Unprocessed images of all blots, gels and TLC plates

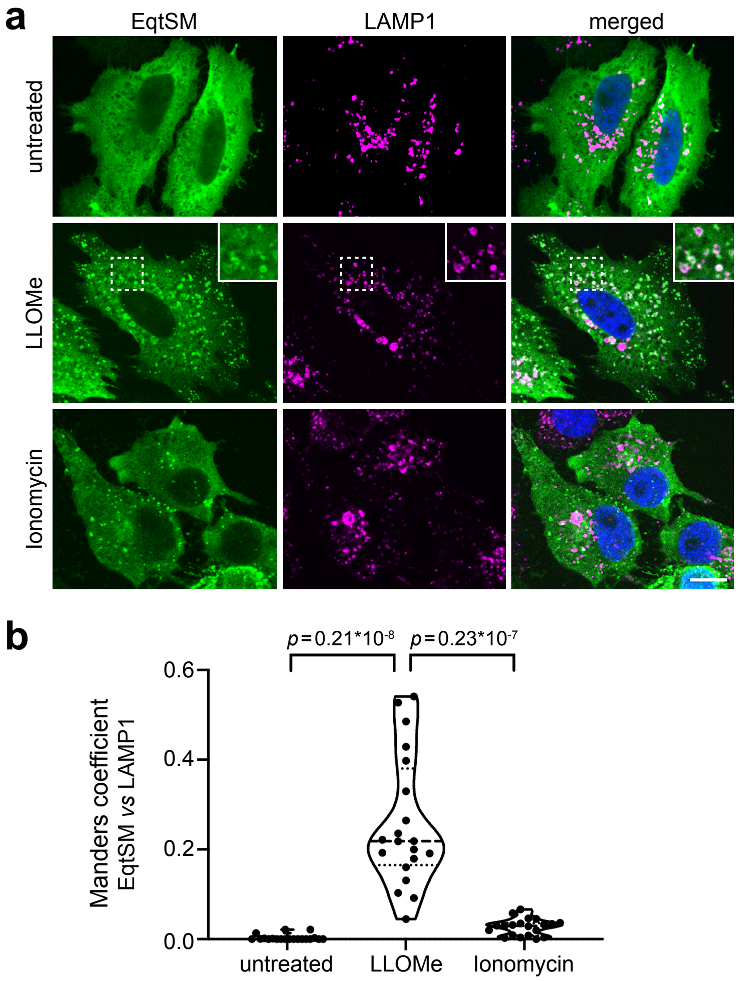


**Supplementary Fig. 1. EqtSM-positive puncta in LLOMe-treated cells co-localize with LAMP1.**

(**a**) HeLa cells expressing GFP-tagged EqtSM (*green*) were treated with LLOMe (1 mM, 20 min) or ionomycin (5 µM, 20 min), fixed, immunostained with an antibody against LAMP1 (*magenta*), counterstained with DAPI (*blue*), and then imaged by confocal fluorescence microscopy. Scale bar, 10 µm. (**b**) Manders' correlation coefficients of fluorescence-intensity-based colocalization of GFP-tagged EqtSM with LAMP1 was calculated from confocal sections of cells treated as in (a). $n$ = 20 cells per condition. For each violin plot, the middle line denotes the median, and the top and bottom lines indicate the 75th and 25th percentile. $p$ values were calculated by unpaired two-tailed t-test. Source data are provided as a Source Data file.

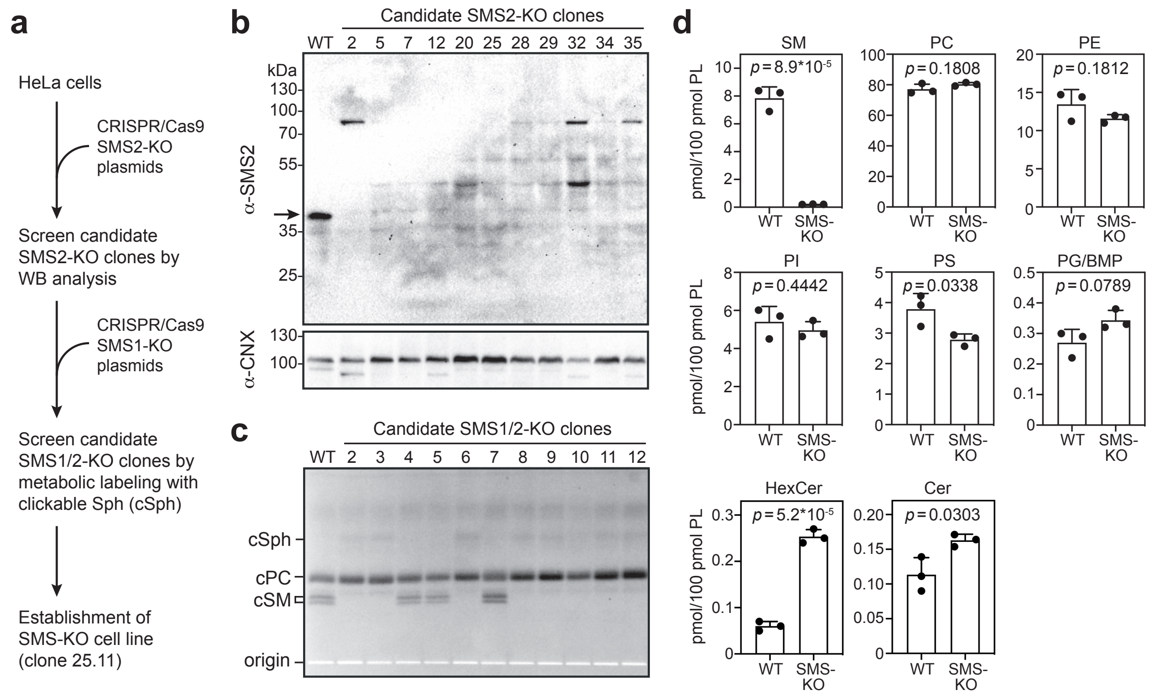


**Supplementary Fig. 2. Generation and characterization of SMS-KO cells.**

(**a**) Schematic outline of SMS1/2 double-KO strategy in HeLa cells. (**b**) SMS2 removal in candidate SMS2-KO clones was verified by immunoblot analysis using antibodies against SMS2 and calnexin (CNX). Migration of immunoreactive SMS2 protein from wildtype cell extracts (WT) is marked by an arrow. (**c**) To verify complete disruption of SM biosynthesis, candidate SMS1/2-KO clones were metabolically labeled with a clickable sphingosine analogue (4 uM, 16 h). Total lipids were extracted, click-reacted with 3-azido-7-hydroxycoumarin, separated by TLC, and analyzed by fluorescence detection. cSph, coumarin-labeled sphingosine; cPC, coumarin-labeled phosphatidylcholine; cSM, coumarin-labeled sphingomyelin. (**d**) Levels of various lipid classes in total lipid extracts from wildtype (WT) and SMS-KO cells (clone 25.11) were determined by LC-MS/MS and expressed in pmol per 100 pmol of total phospholipid analyzed. SM, sphingomyelin; PC, phosphatidylcholine; PE, phosphatidylethanolamine; PI, phosphatidylinositol; PS, phosphatidylserine; PG, phosphatidylglycerol; HexCer, hexosylceramide; Cer. ceramide. Data are means ± SD. $n = 3$ independent experiments. $p$ values were calculated by unpaired two-tailed t-test.

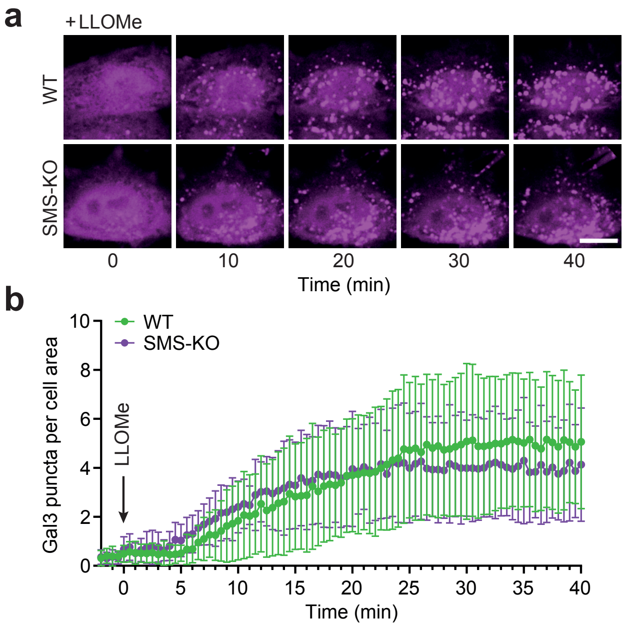


**Supplementary Fig. 3. SM is dispensable for Gal3 recruitment to LLOMe-damaged lysosomes.**

(**a**) Time-lapse fluorescence micrographs of wildtype (WT) or SMS-KO HeLa cells expressing mCherry-tagged galectin-3 (Gal3) and treated with 1 mM LLOMe for the indicated time. Scale bar, 10 μm. (**b**) Time-course plotting Gal3-positive puncta per 100 $\mu m^2$ cell area in cells treated as in (a). Data are means ± SD. *n* = 9 cells for WT, 8 cells for SMS-KO.

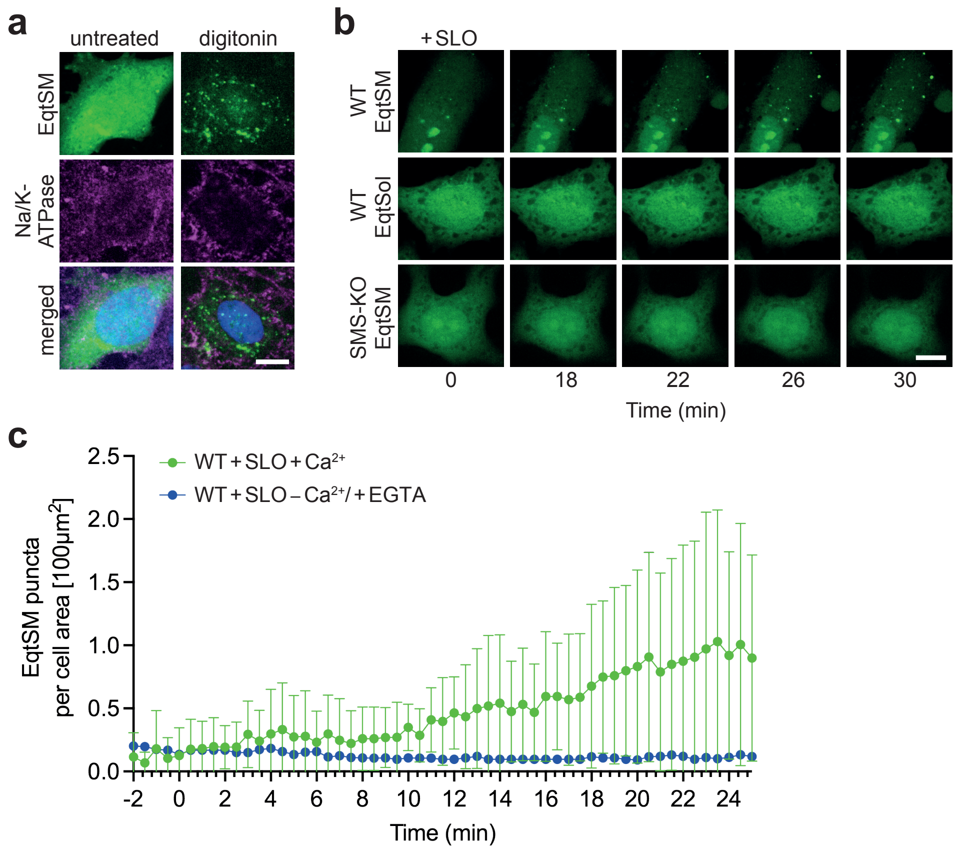


**Supplementary Fig. 4. EqtSM is recruited to the plasma membrane damaged by pore-forming agents.** (**a**) HeLa cells expressing GFP-tagged EqtSM were incubated in the absence or presence of 250 µM digitonin for 1 min. Next, cells were washed twice, incubated at 37°C for 3 min, fixed, immunostained with antibodies against Na/K-ATPase and imaged by confocal fluorescence microscopy. Scale bar, 10 µm. (**b**) Time-lapse fluorescence micrographs of wildtype (WT) or SMS-KO HeLa cells expressing GFP-tagged EqtSM or EqtSol and treated with 1500 U/ml SLO for the indicated time. Scale bar, 10 µm. (**c**) Time-course plotting EqtSM-positive puncta per 100 µm cell area in wildtype (WT) HeLa cells treated with 1500 U/ml SLO for the indicated time in medium containing (+$Ca^{2+}$) or lacking $Ca^{2+}$ (–$Ca^{2+}$/+EGTA). Data are means ± SD. *n* = 6 cells for WT + SLO + $Ca^{2+}$, 7 cells for WT + SLO – $Ca^{2+}$/+ EDTA.

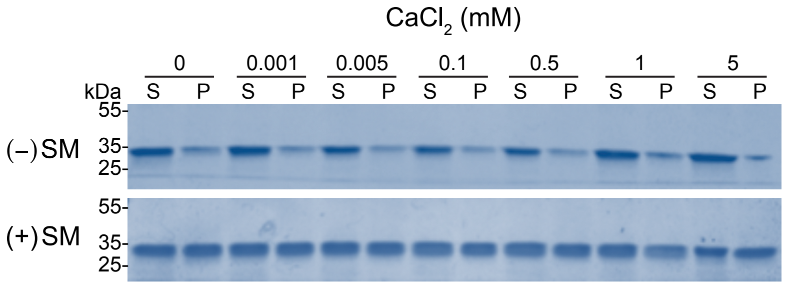


**Supplementary Fig. 5. EqtSM binding to SM-containing liposomes is $Ca^{2+}$-independent.**

Purified recombinant EqtSM protein was incubated with liposomes containing 20 mol% SM (+SM) or a compensatory amount of phosphatidylcholine (−SM) in the presence of the indicated concentration of $CaCl_2$. After 5 min at 37ºC, liposomes were collected by high-speed centrifugation and the bound/pellet (P) and unbound/supernatant (S) fractions were analyzed by to SDS-PAGE and Coomassie blue-staining.

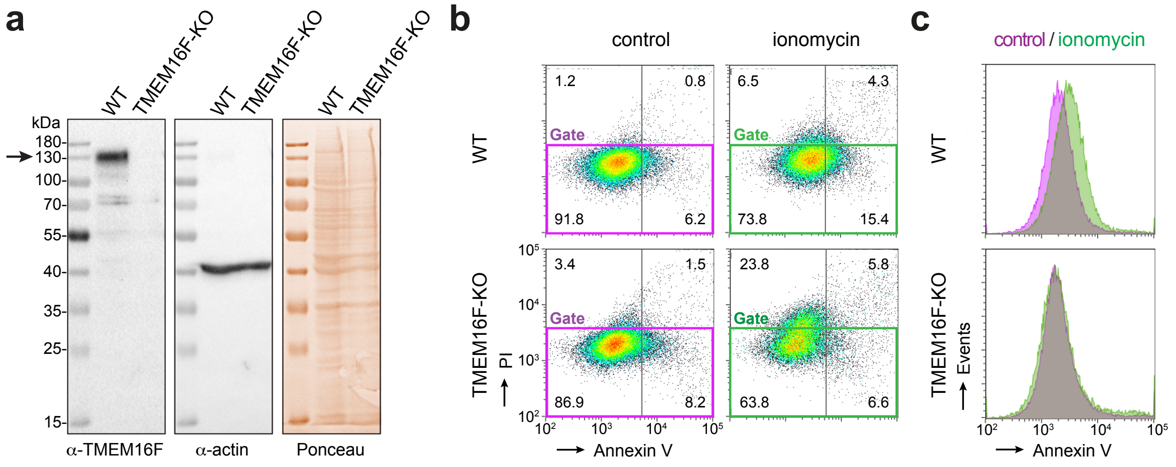


**Supplementary Fig. 6. Characterization of TMEM16F-KO cells.**

(**a**) HeLa cells lacking TMEM16F were created by CRISPR/Cas9. Loss of TMEM16F was confirmed by immunoblot analysis with antibodies against TMEM16F and β-actin. Migration of the TMEM16F protein is marked by an arrow. Data shown are representative of two independent experiments. (**b**) Wildtype (WT) and TMEM16F-KO HeLa cells were incubated in the absence or presence of 15 µM ionomycin or 0.1% (v/v) DMSO (control) for 10 min, stained with annexin V and propidium iodine (PI), and then analyzed by flow cytometry. Representative dot plots are shown. Gates were set to include the majority of PI-negative cells. (**c**) Histograms of annexin V staining of PI-negative cells treated as in (b).

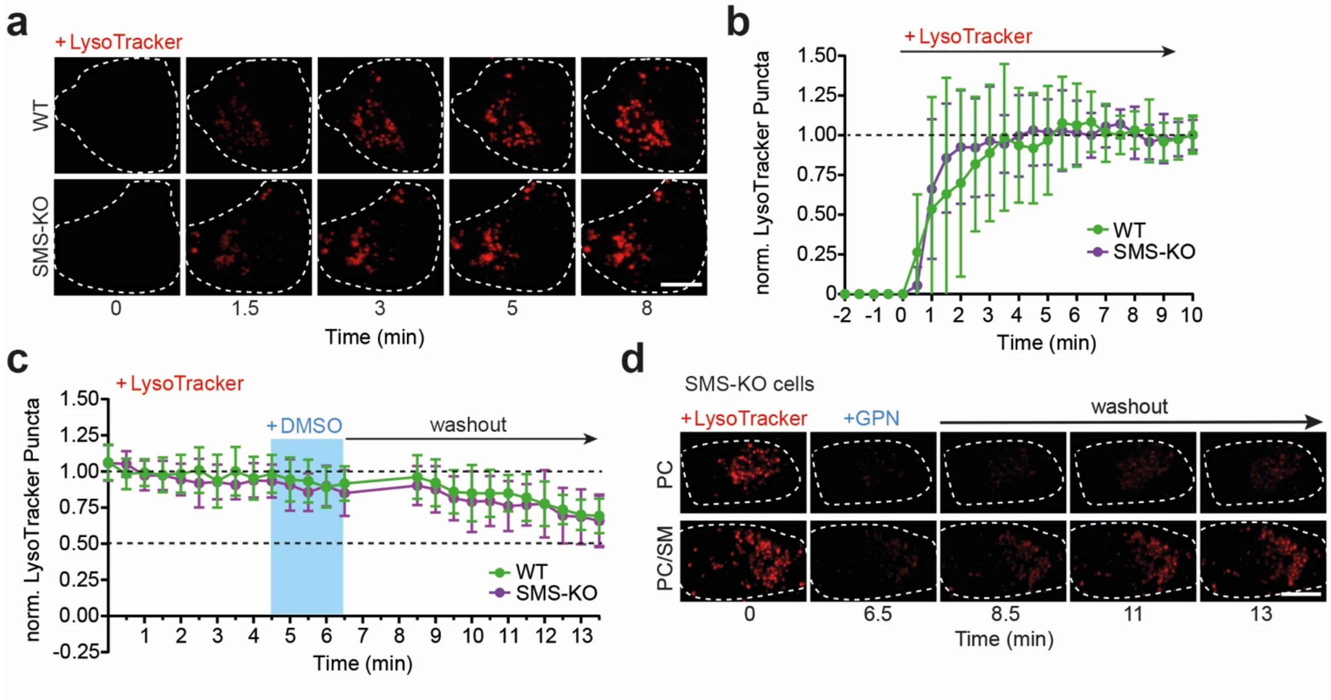


**Supplementary Fig. 7. Wildtype and SMS-KO cells display similar LysoTracker labeling kinetics.**

(**a**) Time-lapse fluorescence micrographs of wildtype (WT) or SMS-KO HeLa cells incubated with 75 nM LysoTracker for the indicated time. Scale bar, 10 µm. (**b**) Time-course plotting LysoTracker-positive puncta in cells treated as in (b), normalized to the maximal number of puncta. Data are means ± SD. *n* = 15 cells for WT, 14 cells for SMS-KO. (**c**) Time-course plotting LysoTracker-positive puncta in wildtype (WT) or SMS-KO HeLa cells during and after a 2 min-pulse of 0.06% (v/v) DMSO as vehicle control for GPN treatment. Data are means ± SD. *n* = 20 cells for WT, 16 cells for SMS-KO. (**d**) Time-lapse fluorescence micrographs of LysoTracker-labeled SMS-KO HeLa cells during and after a 2 min-pulse of GPN (200 µM) following 16 h preincubation with 160 µM PC or PC/SM (70/30 mol%). Data shown are part of the experiment presented in Fig. 3i, with *n* = 50 cells per experimental condition. Scale bar, 10 µm.

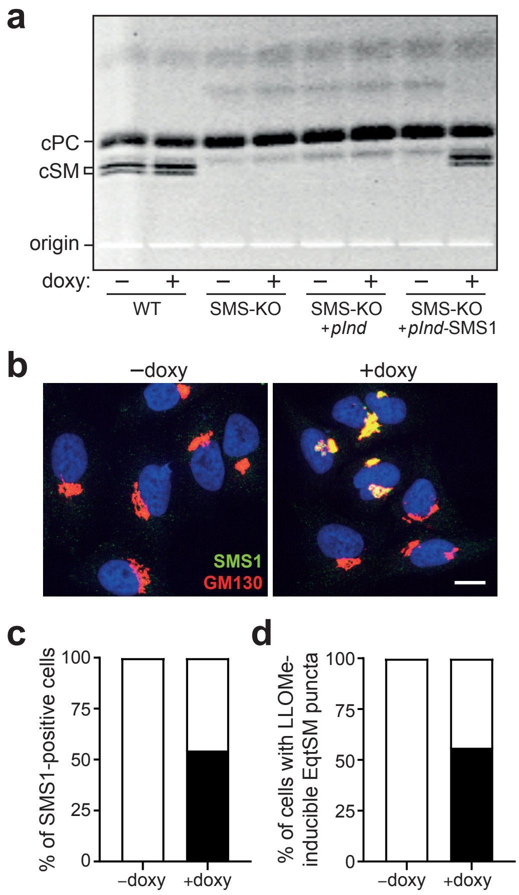


**Supplementary Fig. 8. Characterization of SMS-KO cells transduced with doxycycline-inducible SMS1.** (**a**) Wildtype (WT), SMS-KO or SMS-KO HeLa cells transduced with empty vector (*pInd*) or Flag-tagged SMS1 under control of a doxycycline-inducible promotor (*pInd*-SMS1) were cultured in the absence or presence of 1 µg doxycycline for 32 h. Next, cells were metabolically labeled with a clickable sphingosine analogue for 16 h in the absence (-) or presence (+) of doxycycline. Total lipids were extracted, click-reacted with 3-azido-7-hydroxycoumarin, separated by TLC, and analyzed by fluorescence detection. cPC, coumarin-labeled phosphatidylcholine; cSM, coumarin-labeled sphingomyelin. (**b**) HeLa SMS-KO cells transduced with *pInd*-SMS1 were cultured in the absence or presence of 1 mM doxycycline for 48 h, fixed, stained with antibodies against the Flag-tag (*green*) or the Golgi marker GM130 (*red*) and DAPI (*blue*), and then visualized by confocal microscopy. Scale bar, 10 µm. (**c**) Percentage of SMS-KO cells transduced with *pInd*-SMS1 and treated as in (b) displaying anti-Flag immunostaining. A minimum of 100 cells were analyzed per condition. (**d**) Percentage of SMS-KO cells transduced with *pInd*-SMS1 and expressing GFP-tagged EqtSM displaying GFP-positive puncta in response to GPN (200 mM, 2 min) after 48 h pre-incubation in the presence of absence of 1 µg/ml doxycycline. $n$ = 24 cells analyzed per condition over three independent experiments.

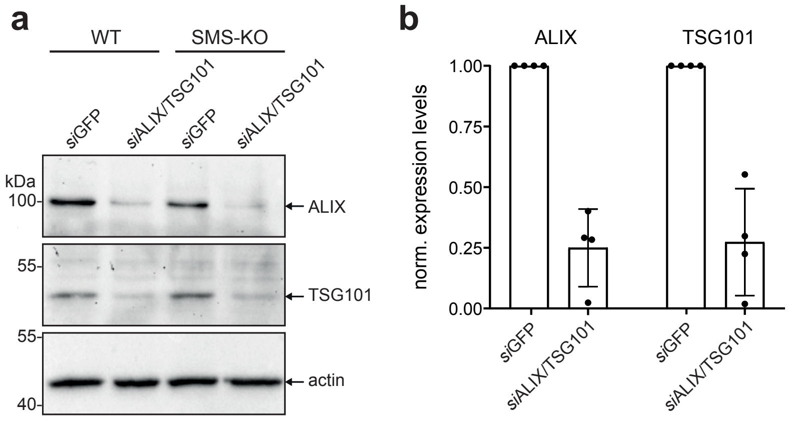


**Supplementary Fig. 9. Validation of siRNA-mediated depletion of ALIX and TSG101.**

(**a**) Wildtype (WT) and SMS-KO HeLa cells were treated with siRNAs targeting GFP (siGFP) or ALIX and TSG101 (siALIX/TSG101) for 72 h, lysed, and then subjected to immunoblot analysis with antibodies against ALIX, TSG101 and β-actin. (**b**) Relative ALIX and TSG101 protein levels in cells treated as in (a) after normalization against actin levels. Data are means ± SD. $n$ = 4 independent experiments.

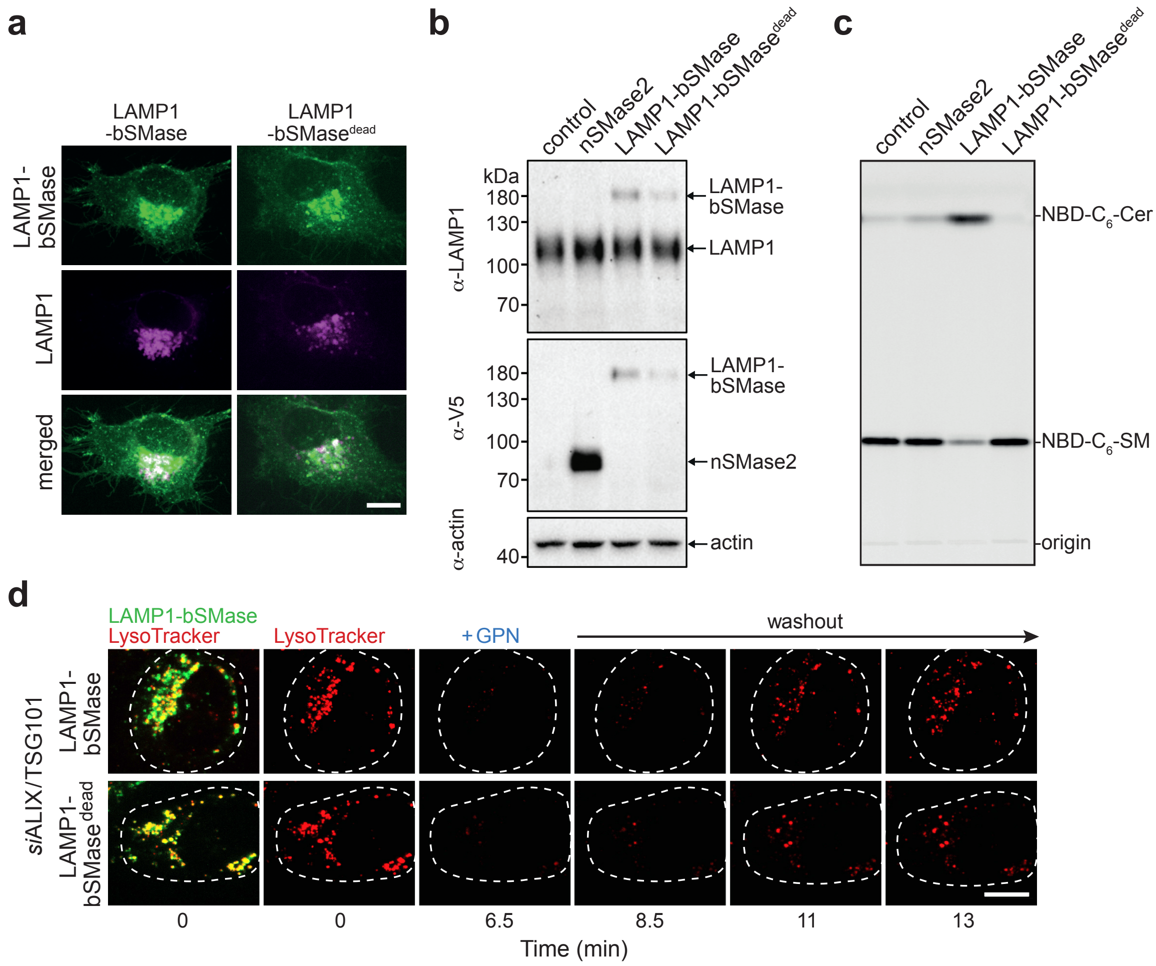


**Supplementary Fig. 10. Characterization of LAMP1-bSMase fusion constructs.**

(**a**) HeLa cells co-transfected with mCherry-tagged LAMP1 (*magenta*) and GFP/V5-tagged LAMP1-bSMase or LAMP1-bSMase$^{dead}$ (*green*) were visualized by confocal fluorescence microscopy. Data shown are representative of two independent experiments. Scale bar, 10 µm. (**b**) HeLa cells transfected with empty vector (control), V5-tagged nSMase2, GFP/V5-tagged LAMP1-bSMase or GFP/V5-tagged LAMP1-bSMase$^{dead}$ were lysed and subjected to immunoblot analysis using antibodies against LAMP1, V5 and β-actin. Data shown are representative of two independent experiments. (**c**) TLC analysis of reaction products formed when lysates of cells treated as in (b) were incubated with 50 µM NBD-C$_6$-SM for 2 h at 37°C. (**d**) Time-lapse fluorescence micrographs of LysoTracker-labeled HeLa cells pretreated with siRNAs targeting ALIX/TSG101 (72 h) and expressing GFP/V5-tagged LAMP1-bSMase or LAMP1-bSMase$^{dead}$ during and after a 2 min-pulse of GPN (200 µM). Data shown are representative of three independent experiments. Scale bar, 10 µm.

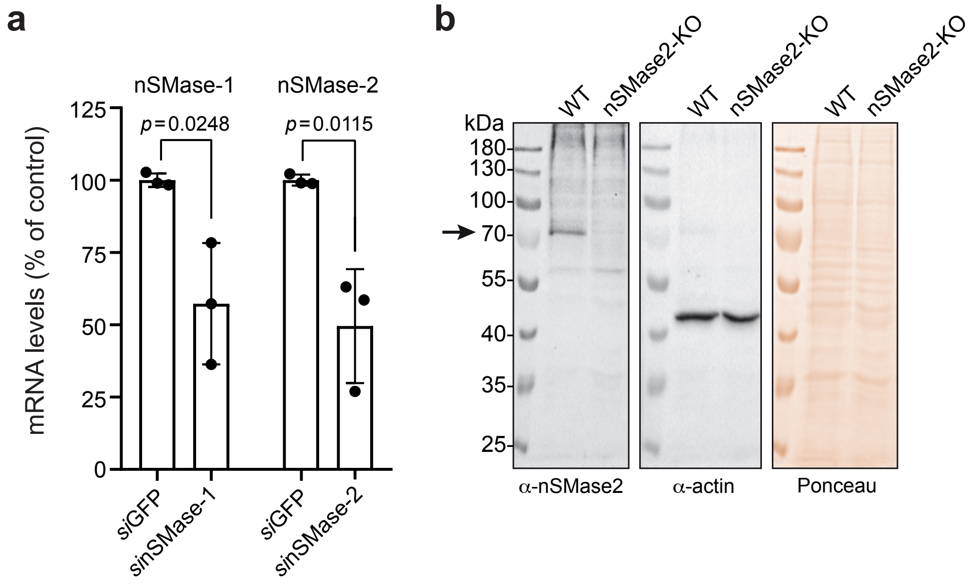


**Supplementary Fig. 11. Validation of siRNA-mediated depletion of nSMase isoforms and CRISPR/Cas9-mediated KO of nSMase2.**

(**a**) HeLa cells were treated with siRNAs targeting GFP, nSMase1 or nSMase2 for 72 h. Total RNA was extracted, converted to cDNA and subjected to RT-qPCR using primers for actin, nSMase1 and nSMase2. Relative levels of nSMase1 and nSMase2 transcripts after normalization against actin are shown. Data are means ± SD. $n = 3$ independent experiments performed in triplicate. $p$ values were calculated by unpaired two-tailed t-test. (**b**) HeLa cells lacking nSMase2 were created by CRISPR/Cas9. Loss of nSMase2 was confirmed by immunoblot analysis with antibodies against nSMase-2 and β-actin following pretreatment of cells with doxorubicin (0.8 µM, 24 h) to boost expression of the corresponding gene. Migration of the nSMase2 protein is marked by an arrow. Data shown are representative of two independent experiments.

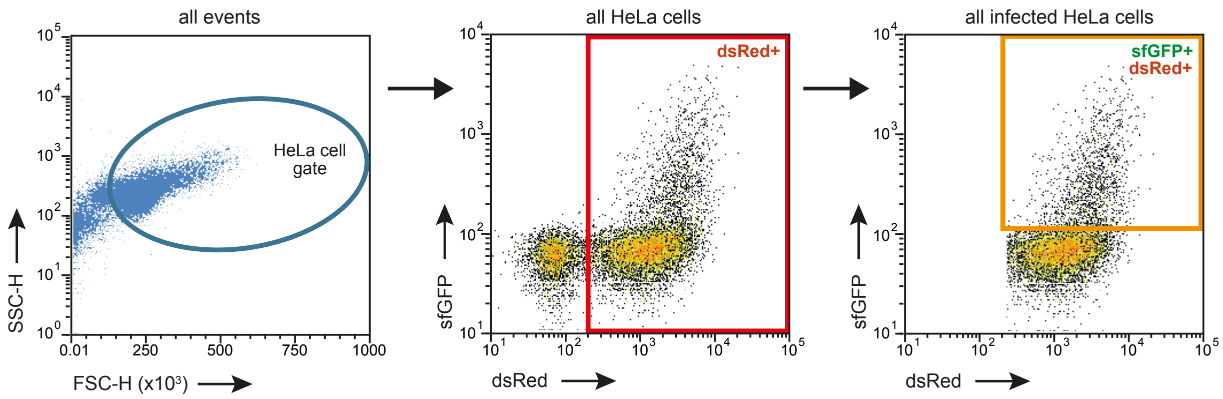


**Supplementary Fig. 12. Gating strategy for flow cytometry analyses of Salmonella infected Hela cells.** HeLa cells were infected with a *Salmonella* strain harboring reporter plasmid p4889 ($P_{EM7}$::*dsred* $P_{uhpT}$::*sfgfp*) for constitutive expression of dsRed and glucose-6-P-induced expression of sfGFP. HeLa cell-sized particles were identified by FSC/SSC gates. For these gated events, dsRed fluorescence was used to gate the *Salmonella*-infected HeLa cell population. The percentage of sfGFP-positive events within the dsRed-positive events was used to calculate the proportion of infected HeLa cells containing *Salmonella* exposed to host cell cytosol within the total population of infected cells.

**Image J Macros**

*Quantification EqtSM-GFP and mCherry-Galectin3 puncta*

1. //set Threshold manually
2. setOption("BlackBackground", false);
3. run("Convert to Mask", "method=Default background=Dark");
4. run("Fill Holes","stack");
5. run("Watershed", "stack");
6. run("Analyze Particles...", "size=0.2-5 circularity=0.50-1.00 show=Outlines display exclude summarize stack");
7. run("Next Slice [>]")
8. //Repeat step 6 and 7 until all time points are analyzed

*Quantification of EqtSM-GFP and LAMP1 overlap*

1. run("Split Channels");
2. //set Threshold manually on EqtSM and LAMP1 channel
3. setOption("BlackBackground", false);
4. setAutoThreshold("Default dark no-reset");
5. //convert applied tresholds for both channels to Mask
6. run("Convert to Mask", "method=Default background=Dark")
7. imageCalculator("AND create", "Channel EqtSM","Channel LAMP1");
8. selectWindow("Result of AND");
9. // Measure area of all and calculate overlap

*Quantification LysoTracker puncta*

1. run("Subtract...", "value=10 stack");
2. setAutoThreshold("Default dark no-reset");
3. setOption("BlackBackground", false);

4. run("Convert to Mask", "method=Default background=Dark");
5. run("Convert to Mask", "method=Default background=Light");
6. run("Watershed", "stack");
7. run("Analyze Particles...", "size=0.2-5 circularity=0.5-1.00 show=Outlines display exclude summarize stack");
8. run("Next Slice [>]")
9. // repeat step 7 and 8 until all time frames have been quantified

*Quantification CHMP4B-GFP and EqtSM-mKate puncta*

1. //perform bleach correction with "exponential fit"
2. //select region that excludes nucleus
3. setOption("BlackBackground", false);
4. run("Convert to Mask", "method=Default background=Dark");
5. run("Fill Holes","stack");
6. run("Analyze Particles...", "size=0.2-5 circularity=0.0-1.00 show=Outlines display exclude summarize stack");
7. run("Next Slice [>]")
8. //Repeat step 6 and 7 until all time points are analyzed

*CHMP4B recruitment / Pre-processing*

1. imageTitle=getTitle();
2. run("Split Channels");
3. selectWindow("C1-"+imageTitle);
4. rename("DAPI");
5. selectWindow("C2-"+imageTitle);
6. rename("Actin");
7. selectWindow("C3-"+imageTitle);
8. rename("CHMP4B");

9. run("Merge Channels...", "c2=[Actin] c1=[CHMP4B] c3=[DAPI] create");
10. run("Z Project...", "projection=[Max Intensity]");
11. run("Split Channels");
12. selectWindow("C1-MAX_Composite");
13. setMinAndMax(200, 2000);
14. run("Subtract...", "value=10");
15. selectWindow("C2-MAX_Composite");
16. setMinAndMax(450, 2200);
17. run("Subtract...", "value=50");
18. selectWindow("C3-MAX_Composite");
19. setMinAndMax(400, 700);
20. run("Merge Channels...", "c1=[C1-MAX_Composite] c2=[C2-MAX_Composite] c3=[C3-MAX_Composite] create");
21. selectWindow("Composite");
22. close();

*CHMP4B recruitment / Quantification*

1. //setTool("freehand");
2. run("Measure");
3. run("Duplicate...", "duplicate");
4. run("Split Channels");
5. close();
6. selectWindow("C1-MAX_Composite-1")
7. setAutoThreshold("Default dark no-reset");
8. //run("Threshold...");
9. setThreshold(1400, 65535);
10. run("Convert to Mask");
11. run("Measure");

12. selectWindow("C2-MAX_Composite-1");

13. close();

# Uncropped Figures

## Supplementary Fig. 2b

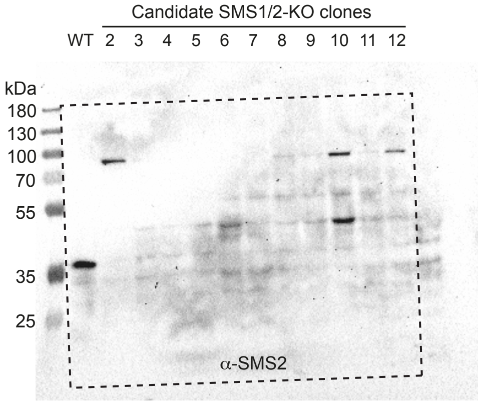


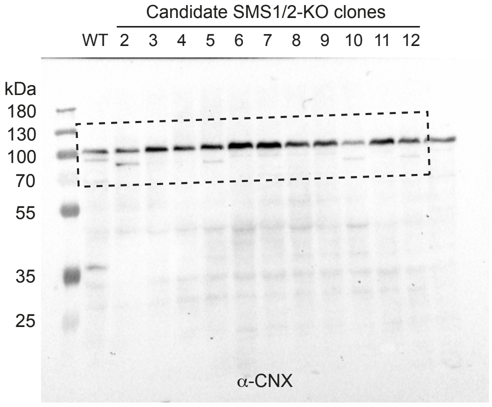


## Supplementary Fig. 2c

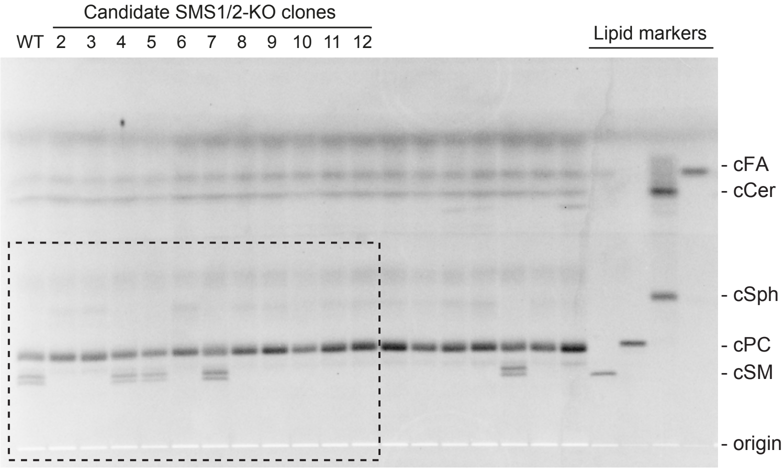


## Supplementary Fig. 5

CaCl$_2$ (mM)

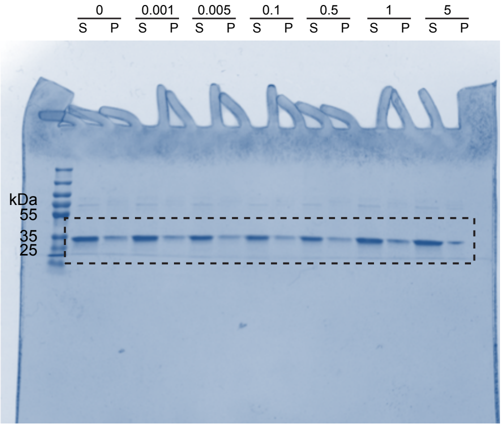


(-)SM

CaCl$_2$ (mM)

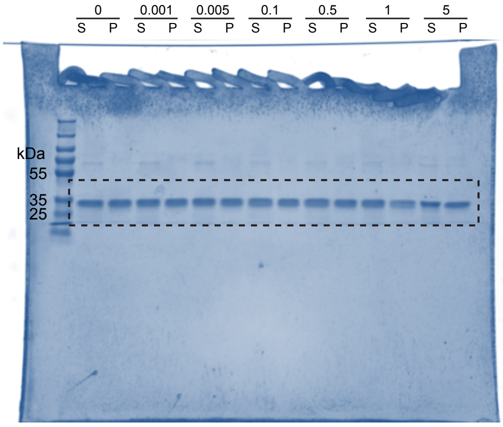


(+)SM

## Supplementary Fig. 8a

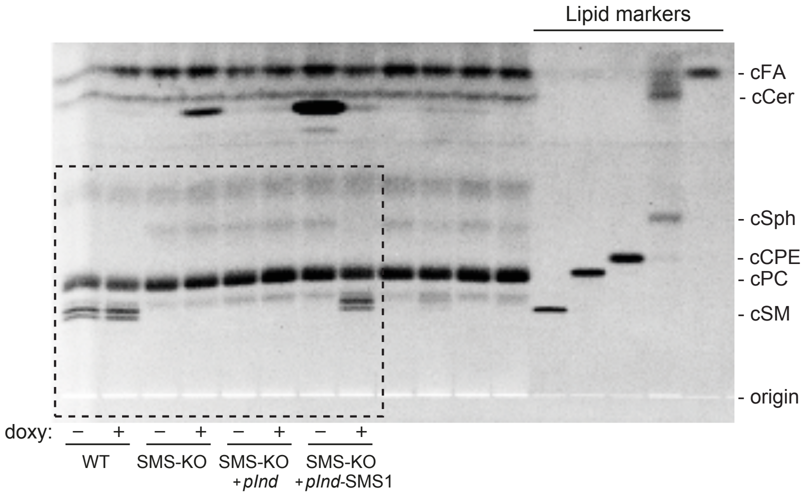


## Supplementary Fig. 9a

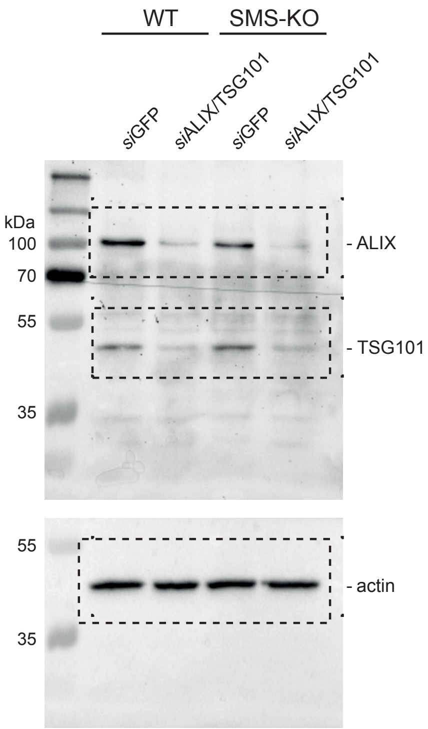

**Supplementary Fig. 10b**

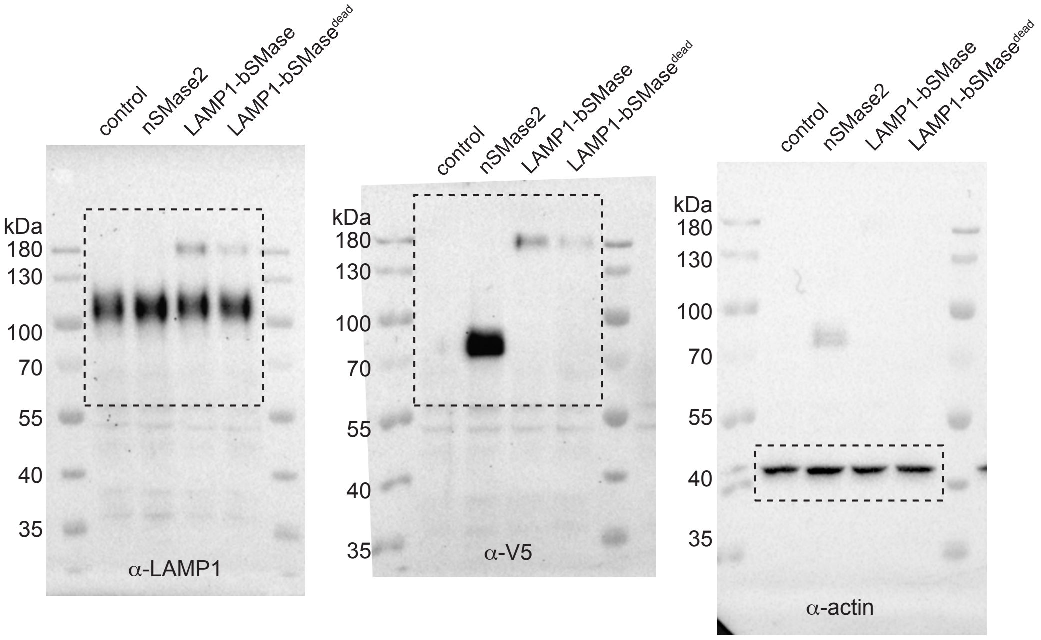


**Supplementary Fig. 10c**

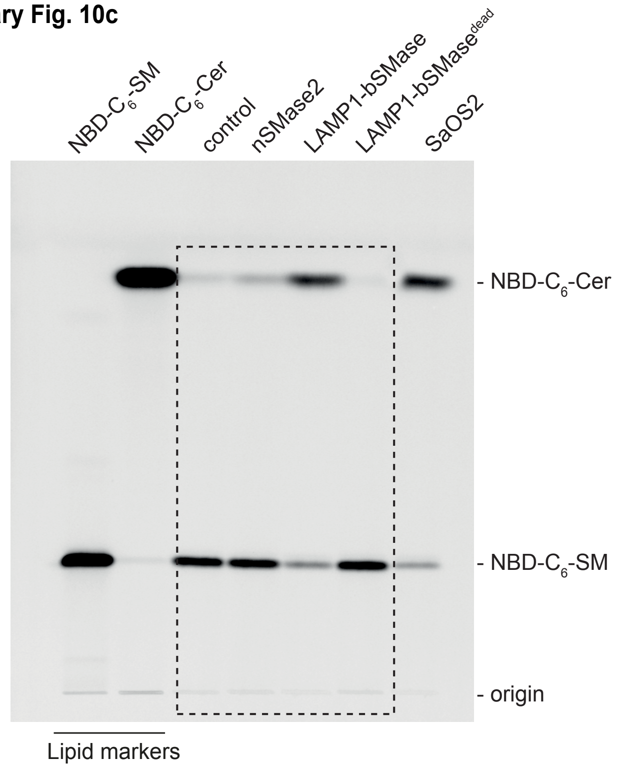

Supplement: Supplementary file 1 — Supplementary Information [file 41467_2022_29481_MOESM1_ESM.pdf]
