# Supplementary material for: Ca2+-activated sphingomyelin scrambling and turnover mediate ESCRT-independent lysosomal repair
Source: Nat Commun. 2022 Apr 6;13:1875. doi: 10.1038/s41467-022-29481-4 (PMC8986845; doi:10.1038/s41467-022-29481-4)
Supplement: Supplementary file 3 — Description of Additional Supplementary Files [file 41467_2022_29481_MOESM3_ESM.pdf]

Description of Additional Supplementary Files

File name: Supplementary Movie 1

Description: Time-lapse images of HeLa cells co-expressing GFP-tagged EqtSM (left, *green*) and mCherry-tagged Gal3 (middle, *magenta*) treated with 1mM LLOMe for the indicated time. Images were captured every 30 s. Scale bar, 10 µm.

File name: Supplementary Movie 2

Description: Time-lapse images of RAW264.7 cells expressing GFP-tagged EqtSM (*green*) infected with mCherry-expressing *M. marinum* (*magenta*). Images were captured every 60 s. Scale bar, 10 µm.

File name: Supplementary Movie 3

Description: Time-lapse images of HeLa cells expressing GFP-tagged EqtSM locally wounded by a brief pulse from a 2-photon laser at high relative intensity (top wound, t = 0 s) and low relative intensity (bottom wound, t = 65 s). Images were captured every 5 s. Scale bar, 10 µm.

File name: Supplementary Movie 4

Description: Time-lapse images of HeLa cells expressing GFP-tagged EqtSM (left, *green*) and labeled with LysoTracker (middle, *red*) during and after a 2 min-pulse of GPN (200 µM). Images were captured every 30 s. Scale bar, 10 µm.

File name: Supplementary Movie 5

Description: Time-lapse images of HeLa cells expressing EqtSM-Halo labeled with silicone rhodamine (*blue*) and infected with *Salmonella* carrying a plasmid for constitutive expression of dsRed (*magenta*) and encoding sfGFP under control of the glucose-6-phosphate (G6P)-inducible promotor P_{uhpT} (*green*). Images were captured every 2 min, starting at 30 min post-infection. Scale bar, 10 µm.

File name: Supplementary Movie 6

Description: Time-lapse images of HeLa cells co-expressing mKate-tagged EqtSM (left, *green*) and eGFP-tagged CHMP4B (middle, *magenta*) treated with 1 mM LLOMe for the indicated time. Images were captured every 10 s. Scale bar, 10 µm.

File name: Supplementary Movie 7

Description: Time-lapse images of HeLa cells co-expressing mKate-tagged EqtSM (left, *green*) and GFP-tagged LAMP1-bSMase (middle, *magenta*) treated with 1 mM LLOMe for the indicated time. Images were captured every 30 s. Scale bar, 10 µm.

File name: Supplementary Movie 8

Description: Time-lapse images of HeLa cells co-expressing mKate-tagged EqtSM (left, *green*) and GFP-tagged LAMP1-bSMase^{dead} (middle, *magenta*) treated with 1 mM LLOMe for the indicated time. Images were captured every 30 s. Scale bar, 10 µm.
