# Supplementary material for: Ca2+-activated sphingomyelin scrambling and turnover mediate ESCRT-independent lysosomal repair
Source: Nat Commun. 2022 Apr 6;13:1875. doi: 10.1038/s41467-022-29481-4 (PMC8986845; doi:10.1038/s41467-022-29481-4)
Supplement: Supplementary file 12 — Reporting Summary [file 41467_2022_29481_MOESM12_ESM.pdf]

nature research

Corresponding author(s): Joost C. M. Holthuis

Last updated by author(s): Feb 16, 2022

Reporting Summary

Nature Research wishes to improve the reproducibility of the work that we publish. This form provides structure for consistency and transparency in reporting. For further information on Nature Research policies, see our Editorial Policies and the Editorial Policy Checklist.

Statistics

For all statistical analyses, confirm that the following items are present in the figure legend, table legend, main text, or Methods section.

n/a	Confirmed	
☐	☒	The exact sample size (*n*) for each experimental group/condition, given as a discrete number and unit of measurement
☐	☒	A statement on whether measurements were taken from distinct samples or whether the same sample was measured repeatedly
☐	☒	The statistical test(s) used AND whether they are one- or two-sided<br>*Only common tests should be described solely by name; describe more complex techniques in the Methods section.*
☒	☐	A description of all covariates tested
☐	☒	A description of any assumptions or corrections, such as tests of normality and adjustment for multiple comparisons
☐	☒	A full description of the statistical parameters including central tendency (e.g. means) or other basic estimates (e.g. regression coefficient) AND variation (e.g. standard deviation) or associated estimates of uncertainty (e.g. confidence intervals)
☐	☒	For null hypothesis testing, the test statistic (e.g. *F*, *t*, *r*) with confidence intervals, effect sizes, degrees of freedom and *P* value noted<br>*Give P values as exact values whenever suitable.*
☒	☐	For Bayesian analysis, information on the choice of priors and Markov chain Monte Carlo settings
☒	☐	For hierarchical and complex designs, identification of the appropriate level for tests and full reporting of outcomes
☒	☐	Estimates of effect sizes (e.g. Cohen's *d*, Pearson's *r*), indicating how they were calculated

Our web collection on statistics for biologists contains articles on many of the points above.

Software and code

Policy information about availability of computer code

Data collection

Laser wounding and time-lapse acquisition were performed using an Olympus model FV3000 laser scanning microscope (Olympus Europa SE & CO. KG) optically coupled to a fs laser system that comprises a regeneratively amplified fs laser (Pharos-HE-20; Light Conversion Inc.) and an optical parametric amplifier (OPA, Orpheus-Twins F; Light Conversion Inc.).

Time-lapse recordings of cells exposed to organelle-damaging drugs or pathogens were performed using a Zeiss Cell Observer Spinning Disc Confocal Microscope equipped with a TempModule S1 temperature control unit, a Yokogawa Spinning Disc CSU-X1a 5000 Unit, a Evolve EMCDD camera (Photonics, Tucson), a motorized xyz-stage PZ-2000 XYZ (Applied Scientific Instrumentation) and an Alpha Plan-Apochromat x 63 (NA 1.46) oil immersion objective.

Cell survival was quantified using an Infinite 200 Pro M-Plex plate reader (Tecan Lifescience).

RT-qPCR reactions were performed on a C1000 Thermal Cycler with a CFX96 Real-Time System (Bio-Rad Laboratories) using MaximaTM SYBRTM Green/ROX 2x qPCR Master Mix (Thermo Fisher Scientific; K0221).

Fluorescent lipids were analyzed using a ChemiDoc XRS+ with UV-transillumination (BioRad Laboratories, USA).

LC-MS/MS was performed using a C30 reverse-phase column (Thermo Acclaim C30, 2.1 × 250 mm, 3 µm, operated at 50° C; Thermo Fisher Scientific) connected to an HP 1100 series HPLC system (Agilent) and a QExactivePLUS orbitrap mass spectrometer (Thermo Fisher Scientific) equipped with a heated electrospray ionization (HESI) probe. MS spectra of lipids were acquired in full-scan/data-dependent MS2 mode.

Flow cytometry of Salmonella-infected HeLa cells was performed using an Attune NxT Cytometer (Thermo Fisher Scientific). Flow cytometry of Annexin V-labeled HeLa cells was performed using a SH800 Cell Sorter (Sony Biotechnology).

Data analysis

Analysis of microscopy images were performed on the original, unmodified data using Fiji Image J2 software (version 2.3.0/1.53f) and Image J

Data analysis

Macros provided in Supplementary Information.

Immunoblot band intensities and fluorescent lipids were quantified using Image Lab 5.2 software (BioRad Laboratories).

RT-qPCR data were analysed using CFX Manager software version 2.1 (Bio-Rad Laboratories).

Flow cytometry data were analyzed using Attune NxT Software version 4.2.0. (Salmonella-infected HeLa cells) or Sony Cell Sorter software version 2.1.5 (Annexin V-labeled HeLa cells).

Lipid mass spec data were analyzed using Lipid Search software version 4.1 (MKI, Tokyo, Japan).

For manuscripts utilizing custom algorithms or software that are central to the research but not yet described in published literature, software must be made available to editors and reviewers. We strongly encourage code deposition in a community repository (e.g. GitHub). See the Nature Research guidelines for submitting code & software for further information.

Data

Policy information about availability of data

All manuscripts must include a data availability statement. This statement should provide the following information, where applicable:

- Accession codes, unique identifiers, or web links for publicly available datasets
- A list of figures that have associated raw data
- A description of any restrictions on data availability

All data generated or analyzed during this study are included in the manuscript and supporting files. The source data underlying Figs. 1b, 1d, 1h, 1f, 2b, 2d, 2f, 2g, 3c, 3e, 3g, 3i, 3j-l, 4d, 5b, 5d, 6c-e, 7a-h and Supplementary Figs. 1b, 2d, 3b, 4c, 7b, 7c, 9 and 11 are provided as a Source Data file. Uncropped scans of immunoblots, gels and TLC plates are provided in Supplementary Information.

Field-specific reporting

Please select the one below that is the best fit for your research. If you are not sure, read the appropriate sections before making your selection.

☒ Life sciences ☐ Behavioural & social sciences ☐ Ecological, evolutionary & environmental sciences

For a reference copy of the document with all sections, see nature.com/documents/nr-reporting-summary-flat.pdf

Life sciences study design

All studies must disclose on these points even when the disclosure is negative.

Sample size	Sample size for each experiment is indicated in the figure and in the corresponding source data, provided as a Source Data file. No statistical method was used for sample predetermination. The sample size was determined based on other studies with similar methodologies (doi:10.1126/science.aar5078; doi:10.15252/embj.201899753; doi:10.1016/j.cub.2020.05.083) and the author's experience of what is necessary to generate a compelling and meaningful result.
Data exclusions	No data were excluded from the analyses in the experiments.
Replication	Except for some supportive experiments for which the outcome was clear cut and verified by complementary approaches (i.e. Supplementary Figs. 2b, 2c, 5, 6b, 8a and 10c), each experiment was repeated at least once with similar results, using independent experimental samples and statistical tests as specified in the figure legends. Source data with sample sizes, number of technical and/or biological replicates, means, standard deviations and calculated p values (where applicable) are provided in the Source Data file for Figs. 1b, 1d, 1h, 1f, 2b, 2d, 2f, 2g, 3c, 3e, 3g, 3i, 3j-l, 4d, 5b, 5d, 6c-e, 7a-h and Supplementary Figs. 1b, 2d, 3b, 4c, 7b, 7c, 9 and 11.
Randomization	Plates or dishes with cultured cells were randomly assigned to experimental groups. Microscope image acquisition was performed randomly.
Blinding	No blinding was done in this study. Virtually all the data are quantitative. Most measurements were made using a machine and not easily subject to operator bias.

Reporting for specific materials, systems and methods

We require information from authors about some types of materials, experimental systems and methods used in many studies. Here, indicate whether each material, system or method listed is relevant to your study. If you are not sure if a list item applies to your research, read the appropriate section before selecting a response.

Materials & experimental systems

n/a	Involved in the study
☐	☒ Antibodies
☐	☒ Eukaryotic cell lines
☒	☐ Palaeontology and archaeology
☒	☐ Animals and other organisms
☒	☐ Human research participants
☒	☐ Clinical data
☒	☐ Dual use research of concern

Methods

n/a	Involved in the study
☒	☐ ChIP-seq
☐	☒ Flow cytometry
☒	☐ MRI-based neuroimaging

Antibodies

Antibodies used

Primary antibodies
Rabbit polyclonal anti-TMEM16F (Sigma-Aldrich; HPA038958; IB 1:1000)
Mouse monoclonal anti-SMS2 [7D10] (Santa Cruz; sc-293384; IB 1:1000)
Mouse monoclonal anti-LAMP1 [H4A3] (Santa Cruz, sc-20011; IF 1:200)
Rabbit polyclonal anti-CHMP4B (Proteintech; 13683-1-AP; IF 1:300)
Mouse monoclonal anti-ALIX [3A9] (Biolegend; 634501; IB 1:1000)
Mouse monoclonal anti-Actin (Sigma-Aldrich; A1978; IF 1:1200; IB 1:10,000)
Rabbit monoclonal anti-Na/K-ATPase [EP1845Y] (Abcam; ab-76020; IF 1:600)
Mouse monoclonal anti-TSG101 [C-2] (Santa Cruz; sc-7964; IB 1:1000)
Mouse monoclonal anti-V5 [R960-25] (Invitrogen; r96025; IF 1:400; IB 1:1000)
Rabbit polyclonal anti-GFP (Novus Biologicals, NB600-303; IF 1:250)

Secondary antibodies
HRP-conjugated goat anti-mouse IgG (Thermo Fisher Scientific; 31430; IB 1:5000)
HRP-conjugated goat anti-rabbit IgG (Thermo Fisher Scientific; 31460; IB 1:5000)
Cyanine Cy™2-conjugated donkey anti-mouse IgG (Jackson ImmunoResearch Laboratories, 715-225-150; IF 1:400)
Cyanine Cy™2-conjugated donkey anti-rabbit IgG (Jackson ImmunoResearch Laboratories, 711-225-152; IF 1:400)
Cyanine Cy™3-conjugated donkey anti-rabbit IgG (Jackson ImmunoResearch Laboratories, 715-165-152; IF 1:400)
Cyanine Cy™3-conjugated donkey anti-mouse IgG (Jackson ImmunoResearch Laboratories, 715-165-150; IF 1:400)

Validation

Primary antibodies / validation statements from the manufacturers:

Rabbit polyclonal anti-TMEM16F (Sigma-Aldrich; HPA038958)
https://www.sigmaaldrich.com/NL/en/product/sigma/hpa038958

Mouse monoclonal anti-SMS2 [7D10] (Santa Cruz; sc-293384)
https://www.scbt.com/p/sms2-antibody-7d10

Mouse monoclonal anti-LAMP1 [H4A3] (Santa Cruz, sc-20011)
https://datasheets.scbt.com/sc-20011.pdf

Rabbit polyclonal anti-CHMP4B (Proteintech; 13683-1-AP)
https://www.ptglab.com/Products/CHMP4B-Antibody-13683-1-AP.htm

Mouse monoclonal anti-ALIX [3A9] (Biolegend; 634501)
https://www.biolegend.com/en-us/products/purified-anti-alix-antibody-4469?GroupID=BLG14736

Mouse monoclonal anti-Actin (Sigma-Aldrich; A1978)
https://www.sigmaaldrich.com/NL/en/product/sigma/a1978

Rabbit monoclonal anti-Na/K-ATPase [EP1845Y] (Abcam; ab-76020)
https://www.abcam.com/sodium-potassium-atpase-antibody-ep1845y-plasma-membrane-loading-control-ab76020.html

Mouse monoclonal anti-TSG101 [C-2] (Santa Cruz; sc-7964)
https://datasheets.scbt.com/sc-7964.pdf

Mouse monoclonal anti-V5 [R960-25] (Invitrogen; r96025)
https://www.thermofisher.com/antibody/product/V5-Tag-Antibody-Monoclonal/R960-25

Rabbit polyclonal anti-GFP (Novus Biologicals, NB600-303)
https://www.novusbio.com/products/gfp-antibody_nb600-303

Secondary antibodies / validation statements from the manufacturers:

HRP-conjugated goat anti-mouse IgG (Thermo Fisher Scientific; 31430)
https://www.thermofisher.com/antibody/product/Goat-anti-Mouse-IgG-H-L-Secondary-Antibody-Polyclonal/31430

HRP-conjugated goat anti-rabbit IgG (Thermo Fisher Scientific; 31460)
https://www.thermofisher.com/antibody/product/Goat-anti-Rabbit-IgG-H-L-Secondary-Antibody-Polyclonal/31460

Cyanine Cy™2-conjugated donkey anti-mouse IgG (Jackson ImmunoResearch Laboratories, 715-225-150)
https://www.jacksonimmuno.com/catalog/products/715-225-150

Cyanine Cy™2-conjugated donkey anti-rabbit IgG (Jackson ImmunoResearch Laboratories, 711-225-152)
https://www.jacksonimmuno.com/catalog/products/711-225-152

Cyanine Cy™3-conjugated donkey anti-rabbit IgG (Jackson ImmunoResearch Laboratories, 715-165-152)
https://www.jacksonimmuno.com/catalog/products/715-165-152

Cyanine Cy™3-conjugated donkey anti-mouse IgG (Jackson ImmunoResearch Laboratories, 715-165-150)
https://www.jacksonimmuno.com/catalog/products/715-165-150

Eukaryotic cell lines

Policy information about cell lines

Cell line source(s)

HeLa cells, ATTC, CCL-2; RAW264.7 macrophages, ATCC, TIB-71; Hek293T cells, ATTC, CRL-3216; HeLa CHMP3-KO cells have been previously described in Ref. 28; HeLa cell-line stably expressing CHMP4B-eGFP was kindly provided by Anthony Hyman (Max Planck Institute for Molecular Cell Biology and Genetics, Dresden, DE) and previously described in Ref. 47.

Authentication

Cell lines were routinely examined for their morphology and analyzed for their characteristic protein expression profiles.

Mycoplasma contamination

All cell-lines were free of mycoplasma contaminations as determined routinely by DAPI staining or PCR assay.

Commonly misidentified lines
(See ICLAC register)

No commonly misidentified cell lines were used.

Flow Cytometry

Plots

Confirm that:

☒ The axis labels state the marker and fluorochrome used (e.g. CD4-FITC).

☒ The axis scales are clearly visible. Include numbers along axes only for bottom left plot of group (a 'group' is an analysis of identical markers).

☒ All plots are contour plots with outliers or pseudocolor plots.

☒ A numerical value for number of cells or percentage (with statistics) is provided.

Methodology

Sample preparation

Flow cytometry of Salmonella-infected HeLa cells (Fig. 4c): cells were incubated in Opti-MEM containing 100 µg/ml gentamicin (AppliChem, A1492) for 1 h. Afterwards, cells were maintained in Opti-MEM with a reduced gentamicin concentration of 10 µg/ml for the rest of the experiment. At 3 h post-infection, cells were washed twice with PBS and detached by incubation in 300 ul of Accutase (Sigma-Aldrich, A6964) for 10 min at 37°C. Upon addition of an equal volume of PBS, cells were collected by centrifugation at 500 x g for 10 min and then incubated in 250 µl Opti-MEM containing 200 µg/ml rifampicin (Sigma-Aldrich, R7382) and 200 µg/ml chloramphenicol (Sigma-Aldrich, C1919) to block bacterial transcription and translation but allow maturation of newly synthesized fluorescence proteins for 30 min at 37°C prior to flow cytometry.

Flow cytometry of Annexin V-labelled HeLa cells (Suppl. Fig. 6b): cells were detached using trypsin, taken up in DMEM containing 10% FBS, washed in PBS and resuspended in Annexin V Binding Buffer (Biolegend, no. 422201) and then incubated in the presence of 15 µM ionomycin or 0.1% (v/v) DMSO for 10 min at 37°C in 5% CO2. Next, APC-Annexin V (Biolegend, no. 640920; 5 µl in 100 µl Binding Buffer) and propidium iodide (5 µg/ml; Sigma Aldrich, P4170) were added and cells were incubated for 10 min at RT. After addition of 400 µl Annexin V Binding Buffer, cells were cooled on ice prior to flow cytometry.

Instrument

Salmonella-infected HeLa cells were analyzed using an Attune NxT Cytometer (Thermo Fisher Scientific).
Annexin V-labelled HeLa cells were analyzed using a SH800 Cell Sorter (Sony Biotechnology).

Software

Flow cytometry data generated with Attune NxT Cytometer were analyzed using Attune NxT Software version 4.2.0.
Flow cytometry data generated with SH800 Cell Sorter were analyzed using Sony Cell Sorter software version 2.1.5.

Cell population abundance

For flow cytometry of Salmonella-infected HeLa cells, at least 10.000 infected (dsRed-positive) cells were gated to calculate the proportion containing host cytosol-exposed Salmonella (dsRed- and sfGFP-positive).

Gating strategy

For flow cytometry of Salmonella-infected HeLa cells, host cells were infected with a Salmonella strain harboring reporter plasmid p4889 (PEM7::dsred PuhpT::sfgfp) for constitutive expression of dsRed and glucose-6-P-induced expression of sfGFP. HeLa cell-sized particles were identified by FSC/SSC gates. For these gated events, dsRed fluorescence was used to gate the Salmonella-infected HeLa cell population. The percentage of sfGFP-positive events within the dsRed-positive events was used

April 2020

to calculate the proportion of infected HeLa cells containing Salmonella exposed to host cell cytosol within the total population of infected cells. The corresponding gating strategy is provided in Suppl. Fig. 12.

For flow cytometry of Annexin V-labelled HeLa cells, gates were set to include the majority of PI-negative cells. The corresponding gating strategy is provided in Suppl. Fig. 6b.

☒ Tick this box to confirm that a figure exemplifying the gating strategy is provided in the Supplementary Information.
